# Assessment of planning reproducibility in three-dimensional field-in-field radiotherapy technique for breast cancer: impact of surgery-simulation interval

**DOI:** 10.1038/s41598-020-78666-8

**Published:** 2021-01-15

**Authors:** Dong Soo Lee, Young Kyu Lee, Young Nam Kang, Yong Gyun Won, Seung Hwan Park, Yong Seok Kim, Jeong Soo Kim, Hye Sung Won

**Affiliations:** 1 Department of Radiation Oncology, College of Medicine, The Catholic University of Korea, Seoul, Republic of Korea; 2 Proton Therapy Center, Research Institute and Hospital, National Cancer Center, Goyang, Republic of Korea; 3 AbbVie Biopharmaceutical Company, Seoul, Republic of Korea; 4 Department of Surgery, College of Medicine, The Catholic University of Korea, Seoul, Republic of Korea; 5 Division of Medical Oncology, Department of Internal Medicine, College of Medicine, The Catholic University of Korea, Seoul, Republic of Korea

**Keywords:** Oncology, Physics

## Abstract

The three-dimensional field-in-field (3-D FIF) technique for radiotherapy is an advanced, state-of-the-art method that uses multileaf collimators to generate a homogeneous and conformal dose distribution via segmental subfields. The purpose of this study is to evaluate the dosimetric reproducibility of 3-D FIF plans using the original simulation computed tomography (iCT) scans and re-simulation CT (rCT) scans for whole breast irradiation (WBI) schedule. This study enrolled a total of 34 patients. The study population underwent iCT scans for standard WBI and took rCT scans after 45 Gy of WBI for cone down boost plans. The dosimetric parameters (V_105%_, V_103%_, V_100%_, V_98%_, V_95%_, V_90%_, V_50%_), plan quality indices (conformity index, homogeneity index) and clinical parameters (isocenter-breast axis, isocenter-lung axis, soft tissue volumes within radiation field, lung volumes within radiation field) were assessed. The median time interval from surgery to iCT was 33 days and from iCT to rCT was 35 days. All dosimetric parameters exhibited statistically significant differences between iCT and rCT among cohorts with a surgery-iCT interval of < 60 days. Homogeneity index showed a statistically significant increase from iCT to rCT among all cohorts. Soft tissue volumes (*p* = 0.001) and isocenter-breast axis (*p* = 0.032) exhibited statistically significant differences among cohorts with surgery-iCT interval < 60 days. Regarding the reproducibility of the 3-D FIF WBI plans, significant changes were observed in dosimetric and clinical factors, particularly in study cohorts with a surgery-simulation interval < 60 days. The main contributing factor of these transitions seemed to be the changes in volume of the soft tissue within the WBI field. Further confirmative studies are necessary to determine the most suitable timing and technique for WBI.

## Introduction

Whole breast irradiation (WBI) is the standard adjuvant therapy for early-stage breast cancer following breast conservation surgery (BCS)^1,2^. WBI reduces the risk of local recurrence and improves long-term survival, so it is an essential component of breast cancer treatment^2,3^. Additional benefits of a sequential boost has also been well supported in previous studies^4–6^. Tangential photon beam irradiation has been widely used in WBI, using parallel-opposed fields with physical wedges^7,8^.

With advances in radiation therapy (RT) technologies utilizing multileaf collimators (MLCs), the field-in-field (FIF) technique has become a favored method for tangential WBI^9–11^. The 3-dimensional FIF (3-D FIF) technique offers advantages in the equivalent dose coverage levels for the target volumes while sparing the normal surrounding structures using modern RT techniques. In addition, the 3-D FIF technique enables a reduction in the contralateral breast doses, decreasing the secondary cancer risk of the contralateral breast^9^. The 3-D FIF technique can be implemented in various dose-fractionation schedules of WBI, including conventional fractionation and hypofractionation. Although there are still debatable issues, a hypofractionated WBI schedule is also commonly implemented to treat various stages of breast cancer^12–14^.

During treatment periods of 3–6 weeks, a number of changes can occur in breast cancer tissue and in normal tissue^15,16^ while the same original plans are applied before a re-simulation to boost treatment. Therefore, there is a critical need to assess the reproducibility of dosimetric parameters during treatment periods despite performing daily or weekly image verification.

In the present study, we aimed to evaluate the dosimetric reproducibility of 3-D FIF plans using original simulations of the computed tomography (CT) scans and re-simulation CT scans for conventional WBI schedules. We also sought to evaluate the changes in several clinical factors and tried to determine the optimum conditions for 3-D FIF planning.

## Results

### Study population

The patient demographics and tumor characteristics are summarized in Table 1. The entire study population was female, with mean age of 55.4 years. All patients underwent BCS: 32 (94.1%) lumpectomies and 2 (5.9%) quadrantectomies. The median time interval from surgery to iCT was 33 days (range, 23–186) and from iCT to rCT was 35 days (range, 33–42). The study population was categorized according to the surgery-iCT time interval of < 60 days (23 patients, 67.6%) and ≥ 60 days (11 patients, 32.4%). The delay in the WBI ≥ 60 days after surgery was due to the chemotherapy schedules.

**Table 1:** Baseline patient and tumor characteristics.

Characteristic	N (%)	Characteristic	N (%)
Age (years)		Histology	
Mean ± SD		Ductal carcinoma in situ	4 (11.8)
55.4 ± 10.5		Lobular carcinoma in situ	1 (2.9)
Site		Invasive ductal carcinoma	29 (85.3)
Right	13 (38.2)	Histologic grade	
Left	21 (61.8)	Well differentiated	8 (23.6)
Quadrant		Moderate differentiated	13 (38.2)
Upper outer quadrant	16 (47.1)	Poorly differentiated	13 (38.2)
Upper inner quadrant	5 (14.7)	Resection margin	
Lower outer quadrant	5 (14.7)	Wide (>1mm)	31 (91.2)
Lower inner quadrant	1 (2.9)	Close (≤ 1mm)	3 (8.8)
Central	7 (20.6)	Hormone receptor	
Surgery		Positive	25 (73.5)
Lumpectomy	32 (94.1)	Negative	8 (23.5)
Quadantectomy	2 (5.9)	Unknown	1 (3)
pT stage		HER-2	
T_IS_	5 (14.7)	Positive	6 (17.6)
T1	20 (58.8)	Negative	28 (82.4)
T1a	3 (8.8)	Ki-67	
T1b	4 (11.8)	< 15%	14 (41.2)
T1c	13 (38.2)	≥ 15%	18 (52.9)
T2	9 (26.5)	Unknown	2 (5.9)
pN stage			
N0	28 (82.4)		
N1	5 (14.7)		
Nx	1 (2.9)		

### Baseline distribution of dosimetric parameters, plan quality indices and clinical parameters

Table 2 shows the baseline dosimetric characteristics and statistical comparisons between iCT and rCT. In the normality test, V_105%_ of iCT and rCT, and V_103%_ of rCT indicated a non-normal distribution. Therefore, a Wilcoxon rank sum test was conducted for these parameters. Other parameters showed a normal distribution, and a paired *t*-test was applied. A comparison of the dosimetric parameters showed that all parameters had statistically significant differences between iCT and rCT among the entire population. The measured values increased for V_105%_ and V_103%_ from iCT to rCT, and decreased for all remaining parameters. However, when the study population was categorized according to the surgery-iCT time interval, all parameters exhibited statistically significant differences between iCT and rCT among cohorts with a surgery-iCT interval < 60 days, but only V_105%_ and V_103%_ were significantly different among cohorts with a surgery-iCT interval ≥ 60 days. In plan quality indices, the homogeneity index (HI) of iCT and rCT indicated a non-normal distribution, and the HI values increased from iCT to rCT (*p* = 0.001).

**Table 2:** Dosimetric comparison of volume parameters and plan quality indices in iCT and rCT (categorization according to the surgery–simulation interval).

	iCT, median (range)	rCT, median (range)	*p*-value		iCT, median (range)	rCT, median (range)	*p*-value
V_105%_ (cc)				V_90%_ (cc)			
Total	0 (0–2.8)	21.4 (0–226.9)	< 0.001^a^	Total	1007.1 (432.5–1558.9)	955.7 (505.8–1437.5)	0.009^b^
< 60 days	0 (0–2.8)	18.9 (0–226.9)	< 0.001^a^	< 60 days	1038.2 (645–1558.9)	958 (598.3–1437.5)	0.004^b^
≥ 60 days	0 (0–07)	23.9 (0.7–146.3)	0.003^a^	≥ 60 days	959.1 (432.5–1293.8)	950.8 (505.8–1269.4)	0.887^b^
V_103%_ (cc)				V_50%_ (cc)			
Total	142.4 (0–312.5)	183 (12.1–512.1)	0.004^a^	Total	1413.7 (698–2094)	1318.8 (793.3–1896)	0.011^b^
< 60 days	137.8 (19.6–312.5)	175.9 (12.1–512.1)	0.059^a^	< 60 days	1445.3 (955–2093.9)	1325.9 (909–1896)	0.008^b^
≥ 60 days	146.4 (0–257.4)	201.3 (40.5–368.5)	0.016^a^	≥ 60 days	1321.9 (698–1675.5)	1311.6 (793.3–1735.2)	0.783^b^
V_100%_ (cc)				Conformity index			
Total	540.2 (195.1–915.7)	512 (225–922.9)	0.037^b^	Total	1.4 (1.2–1.8)	1.4 (1–2.2)	0.939^b^
< 60 days	546.5 (298.6–915.7)	474.6 (300.1–922.8)	0.009^b^	< 60 days	1.4 (1.2–1.8)	1.3 (1–2.2)	0.492^b^
≥ 60 days	532.4 (195.1–818.8)	545.7 (225–784.1)	0.656^b^	≥ 60 days	1.5 (1.3–1.8)	1.5 (1.3–1.9)	0.075^b^
V_98%_ (cc)				Homogeneity index			
Total	687.7 (262.3–1119.5)	653 (307.3–1094.7)	0.005^b^	Total	0.17^c^ (0.1–0.8)	0.29^c^ (0.1–1.0)	0.001^a^
< 60 days	689.9 (424.2–1119.5)	645 (399.4–1094.7)	< 0.001^b^	< 60 days	0.18^c^ (0.1–0.8)	0.31^c^ (0.1–1.0)	0.026^a^
≥ 60 days	675.2 (262.3–985.9)	660.9 (307.3–925.7)	0.956^b^	≥ 60 days	0.16^c^ (0.1–0.3)	0.25^c^ (0.1–0.5)	0.014^a^
V_95%_ (cc)							
Total	837.6 (337.5–1301.3)	818.9 (397.4–1263.5)	0.009^b^				
< 60 days	849.0 (536.5–1301.3)	828.1 (493.8–1263.5)	0.002^b^				
≥ 60 days	812.5 (337.5–1141.2)	797.9 (397.4–1065.5)	0.984^b^				

^a^Wilcoxon rank sum test, ^b^Paired t-test, ^c^Mean value.

Table 3 shows the baseline characteristics of clinical parameters and statistical comparisons between iCT and rCT. Among the entire study population, only soft tissue volumes (within RT field) were statistically different between iCT and rCT (*p* = 0.002). Among 34 patients, 25 (73.5%) patients presented decreased values, and the remaining 9 (26.5%) patients exhibited increased values in soft tissue volumes. In total, the median 1247 cc (range, 566.8–2004.8) decreased to 1233 cc (648.2–1882) from iCT to rCT. However, similarly in Table 2, when the study population was categorized according to the surgery-iCT time interval, the soft tissue volumes (*p* = 0.001) and the isocenter-breast axis (*p* = 0.032) exhibited statistically significant differences among cohorts with a surgery-iCT interval < 60 days, but no parameters showed significant differences among cohorts with surgery-iCT interval ≥ 60 days.

**Table 3:** Comparative results of measured clinical parameters in iCT and rCT (categorization according to the surgery–simulation interval) (*p*-value: Paired *t*-test).

	iCT, median (range)	rCT, median (range)	*p*-value
Isocenter-Breast axis (cm)			
Total	6.8 (4.4–8.2)	6.6 (4.3–8.3)	0.154
< 60 days	6.8 (5.5–8.2)	6.5 (5.4–8.3)	0.032
≥ 60 days	6.8 (4.4–7.7)	6.9 (4.3–8.3)	0.968
Isocenter-Lung axis (cm)			
Total	1.8 (1–2.6)	1.8 (0.7–2.9)	0.411
< 60 days	1.7 (1–2.6)	1.7 (0.8–2.9)	0.764
≥ 60 days	2 (1.2–2.3)	1.9 (0.7–2.7)	0.282
Soft tissue (within RT field) (cc)			
Total	1247 (566.8–2004.8)	1233 (648.2–1882)	0.002
< 60 days	1245.9 (870.1–2004.8)	1226.1 (825.7–1882)	0.001
≥ 60 days	1248.1 (566.8–1594.7)	1290.8 (648.2–1603.2)	0.667
Lung (within RT field) (cc)			
Total	100 (24.5–211)	97.4 (14.6–226.4)	0.917
< 60 days	89.6 (24.5–211)	96.4 (14.6–214.5)	0.833
≥ 60 days	114.8 (33.1–150.2)	106.2 (16.3–226.4)	0.903

### Correlation analyses of parameters

In the normality test, all clinical parameters demonstrated a normal distribution. In the correlation analyses, the isocenter-lung axis and lung volume (*p* < 0.001 and *p* < 0.001), and isocenter-breast axis and soft tissue volume (*p* < 0.001 and *p* < 0.001) displayed a statistically significant positive correlation in both iCT and rCT. The isocenter-breast axis and lung volume (*p* = 0.654 and *p* = 0.936), and the isocenter-lung axis and soft tissue volume (*p* = 0.121 and *p* = 0.579) did not show any statistically significant difference in both iCT and rCT. The results are summarized in Table 4.

**Table 4:** Representative results of correlation analyses between clinical parameters.

Parameters	*p*-value
iCT	
Isocenter-Breast axis (cm) vs Lung (within RT field) (cc)	0.654
Isocenter-Lung axis (cm) vs Lung (within RT field) (cc)	< 0.001
Isocenter-Breast axis (cm) vs Soft tissue (within RT field) (cc)	< 0.001
Isocenter-Lung axis (cm) vs Soft tissue (within RT field) (cc)	0.121
rCT	
Isocenter-Breast axis (cm) vs Lung (within RT field) (cc)	0.936
Isocenter-Lung axis (cm) vs Lung (within RT field) (cc)	< 0.001
Isocenter-Breast axis (cm) vs Soft tissue (within RT field) (cc)	< 0.001
Isocenter-Lung axis (cm) vs Soft tissue (within RT field) (cc)	0.579

### Representative case illustrations

**Figure 1:**
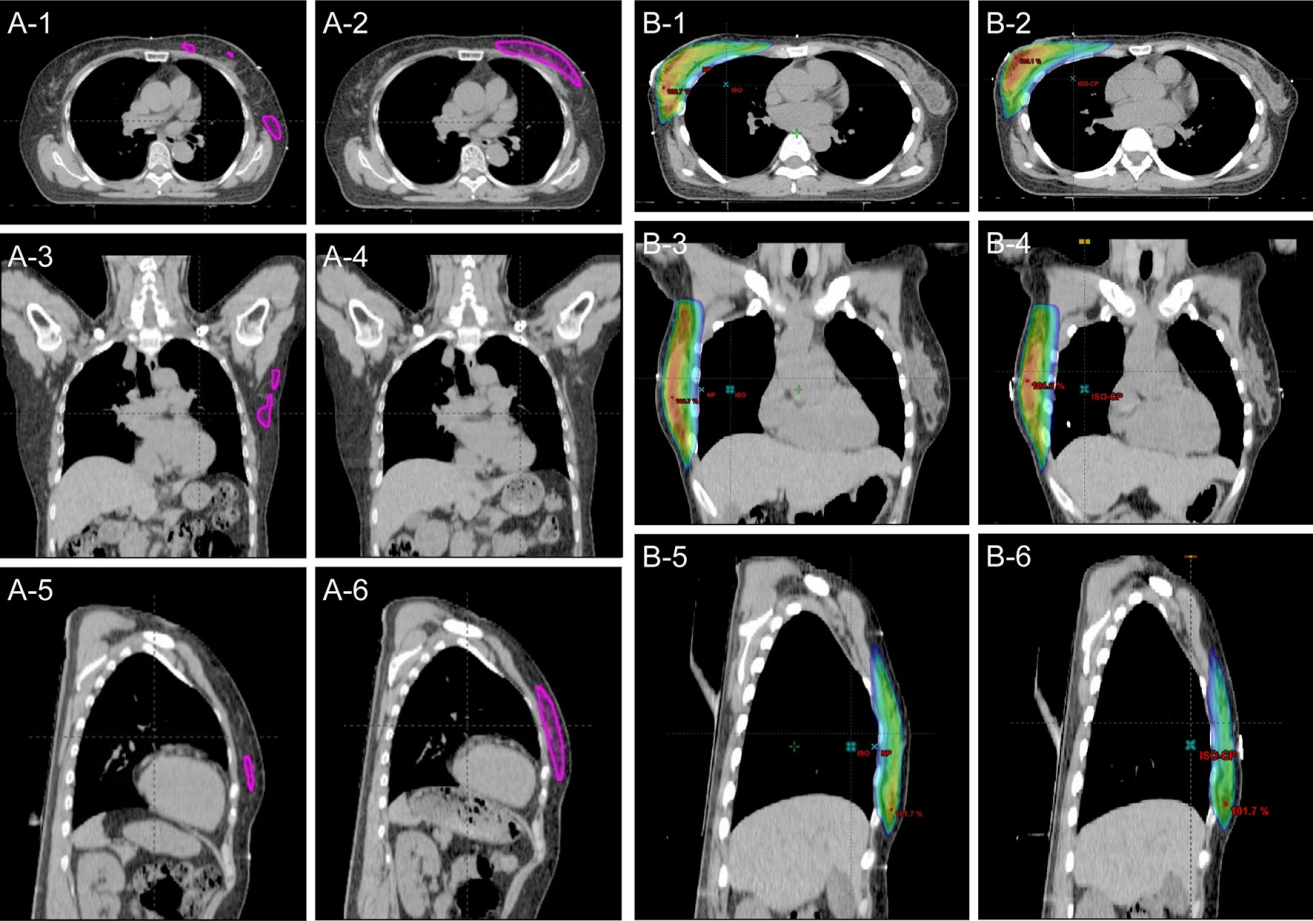
Representative illustration cases. A-1: 103% isodose lines at iCT in patient 1 (axial image), A-2: 103% isodose lines at rCT in patient 1 (axial image), A-3: 103% isodose lines at iCT in patient 1 (coronal image), A-4: 103% isodose lines at rCT in patient 1 (coronal image), A-5: 103% isodose lines at rCT in patient 1 (sagittal image), A-6: 103% isodose lines at rCT in patient 1 (sagittal image), B-1: 95% isodose lines at iCT in patient 2 (axial image), B-2: 95% isodose lines at rCT in patient 2 (axial image), B-3: 95% isodose lines at iCT in patient 2 (coronal image), B-4: 95% isodose lines at rCT in patient 2 (coronal image), B-5: 95% isodose lines at iCT in patient 2 (sagittal image), B-6: 95% isodose lines at rCT in patient 2 (sagittal image).

The representative cases of treated patients are shown in Fig. 1. We can observe the dose distribution changes in the iCT and rCT for each case.

## Discussion

In the current study, we aimed to demonstrate the reproducibility of the actual dosimetric parameters in breast 3-D FIF plans. With a median of 35 days of WBI, measured soft tissue volume (within RT field) was the only statistically different clinical factor among the entire study population. However, when the study population was divided according to the surgery-iCT interval, the results were identical only among cohorts with a surgery-iCT interval < 60 days, and there was no statistically significant difference in the clinical parameters among cohorts with a surgery-iCT interval ≥ 60 days. Similarly, all measured dosimetric parameters (V_105%_, V_103%_, V_100%_, V_98%_, V_95%_, V_90%_, V_50%_) were significantly different between the iCT and rCT in the cohorts with a surgery-iCT interval < 60 days. However, the dosimetric parameters were not significantly different between iCT and rCT, except for V_105%_ and V_103%_, among cohorts with a surgery-iCT interval ≥ 60 days. We also observed that while the overall beam distributions seemed to be analogous, the individual isodose lines formed fairly differently if the same 3-D FIF plans were applied from iCT to rCT. In plan quality indices, only the HI (ideal value = 0) displayed a statistically significant increase from iCT to rCT regardless of the surgery-iCT interval. This was probably a result of the compromise of dosimetric uniformity, particularly by an increase of V_105%_ and V_103%_ in the rCT.

During the treatment periods for WBI, a number of variabilities and uncertainties can be presented, including variabilities in daily set-up and verification systems as well as changes in postoperative seroma or breast deformations^15–19^. Several prior works described changes in factors and their associated dosimetric effects^16,19,20^. The change and effect of a lumpectomy cavity during the course of breast RT has also been widely discussed. In most patients (89%), the seroma volume decreased during the course of partial breast irradiation (PBI), and a median 60% of the seroma volume decreased at the last fraction (10th fraction) of PBI when assessed using a magnetic resonance imaging scan in Jeon et al^15^. The cavity reduction was greatest in the population with large postoperative cavities on the initial CT, as observed by Lee et al^19^. However, the effect of surgery-simulation interval on dosimetric and clinical parameters has not been fully documented to date, and no study reported on the clinical significance and distinctive impact of the time to WBI despite the heterogeneous distribution of surgery-simulation intervals among studies^15,16,19^. Our study results theoretically support that dosimetric and clinical parameter transitions could occur more meaningfully within immediate postoperative healing periods until full tissue resolutions have been established. Among 23 patients with surgery-iCT interval < 60 days, the soft tissue volume decreased in 18 patients (78.3%) and increased in 5 patients (21.7%), and the median 1245.9 cc decreased to 1226.1 cc (*p* = 0.001). On the other hand, among 11 patients with a surgery-iCT interval ≥ 60 days, the soft tissue volume decreased in 7 patients (63.6%) and increased in 4 patients (36.4%) while the median 1248.1 cc increased to 1290.8 cc (*p* = 0.667).

With respect to the breast WBI techniques, the 3-D conformal FIF technique has been recommended as the initial treatment planning approach for WBI in the contemporary era (American Society for Radiation Oncology guideline)^21^. Although a more sophisticated beam delivery technique has been developed to allow for better dose distribution, improved homogeneity, reduced acute reactions and relative sparing of normal organs using intensity-modulated radiotherapy (IMRT), several limitations remain including an increased dose and exposure of the opposite lung and breast, which can increase the risk of second malignancies, requires more expenses, longer treatment-planning and treatment-delivery time, trained personal and devoted quality assurance, as well as ensuring availability of the IMRT facilities^10,22,23^. We also already perceived that daily cone beam CT (CBCT)-based image match and higher doses (monitor unit) generated by IMRT could hypothetically increase the risk of second malignancies^24,25^. Therefore, among various RT delivery techniques in breast WBI, which technique is constantly superior over the others and the standard of care have not been standardized and are debatable^22,26^.

The assessment of reproducibility and the change in the dosimetric parameters in 3-D FIF plans using re-simulation CT scans have not been conducted before, and we have demonstrated that significant dosimetric changes were principally attributed to the soft tissue volume changes, particularly in the early postoperative periods, not by changes in other clinical factors (such as lung volumes within WBI field or other geometric changes) when the same 3-D FIF plans were applied to rCT scans. We could not assure which factors between the surgery-iCT interval or total duration of WBI course would more predominantly affect changes in dosimetric or clinical factors because even in 10 fractions of PBI course, the significant changes were reported in seroma volume^15^ (in this study PBI was started no more than 6-weeks after surgery in the entire study cohorts), and these changes can contribute to the significant dosimetric transitions. From a clinical aspect, the optimal period of initiation of the WBI after surgery is still a debatable issue, and the oncological results have been conflicting among various studies^27–29^.

The strengths of this study are the composition of study cohorts undergoing homogeneous surgical methods, significantly discriminative statistical results and detailed parameter analyses depending on the surgery-simulation intervals. The limitations are a lack of evidence supporting the entire reasons for soft tissue volume changes or deformation during treatment intervals, and the relatively small study sample size. Changes in the soft tissue volumes can arise from various factors, and body weight changes could also contribute to alterations in the breast shape. In addition, the amount of change in the soft tissue volume was comparatively small in relation to the total soft tissue volumes in our study, which was probably due to the measurement process of the soft tissue volumes based on the V_50%_, not fat or fibroglandular tissues. However, this measurement method would be more reliable in terms of minimizing artificial errors conducted by the researchers.

In conclusion, the present study demonstrated significant dosimetric and clinical factor changes in the study population with a surgery-simulation interval < 60 days with respect to the reproducibility of 3-D FIF WBI plans. The main contributing factor of the transition was the soft tissue volume changes within the WBI field. Although our study is partly preliminary, the study results provide informative lessons that dosimetric reproducibility can be considerably impeded within early postoperative periods. The most ideal breast WBI technique, fractionation schedule and initiation timing of WBI remain unknown, and future confirmative studies are needed.

## Materials and methods

### Study design

This study included 34 randomly-designated patients who were diagnosed with early-stage breast cancer (including ductal carcinoma in situ/lobular carcinoma in situ/T1/T2) and underwent BCS followed by postoperative WBI. We obtained Institutional Review Board approval at College of Medicine, the Catholic University of Korea for this clinical investigation (UC15RISE0153) including waiver of informed consent process. The study methods were performed in accordance with the relevant guidelines and regulations by Declaration of Helsinki. WBI was performed using the 3-D FIF technique. All patients underwent simulation CT (iCT) scans for standard WBI and took re-simulation CT (rCT) scans for cone down boost plans with the same positions. As described before, the purpose of this study was to assess the dosimetric reproducibility of the plans. Therefore, the original 3-D FIF plans accomplished at the iCT scans were identically reproduced and applied to rCT scans at the same isocenter. Then, clinical and dosimetric factors and indices were compared to determine which factors could contribute to a difference in the planning parameters.

### Simulation and planning process

All patients underwent iCT and rCT using a CT simulator (SOMATOM Definition AS + , Siemens Medical Solutions USA, Inc.) without contrast enhancement. During the simulation process, the patients were situated on the breast-board in the supine position and both arms were raised above the head using an armrest immobilization device. To preserve the treatment position, the breast-board was fixed to the CT table. CT data were obtained in 3-mm thick slices, covering the entire breast and thorax with normal, free breathing; then, CT datasets were transferred to a Eclipse treatment planning system (ECLIPSE™, version 10; Varian Medical Systems, Palo Alto, CA, USA). The 3-D FIF planning was carried out using the Eclipse treatment planning system in the same manner as in prior works^10,11,30^. Among various FIF techniques^27^, the alternate subfields method was used in the entire patients. In brief, the dose distribution was calculated using the tangential field technique without physical wedges. Then, the MLCs were handled to shield the areas of the breast receiving doses > 105% of the prescription dose by viewing the dose distribution using the beam’s-eye view. The weight of additional subfields using MLCs to reduce hot regions made by the primary tangential fields was approximately 6–10% of the total dose. Finally, after the recalculation process, if hot regions > 107% remained, the aforementioned processes were repeated to obtain an optimal dose distribution. All additional subfields were set not to shield the field isocenter. We tried to adequately cover all postoperative tumor beds including seroma and sought to encompass the chest wall above 95% of the prescription dose. Thus, minor hot areas (V_105%_) below 3-cc were allowed to fulfill the suitable dose distribution profile. The axillary lymph node (LN) stations were covered depending on the pathological N stage and the LN biopsy status.

### Dosimetric parameters, plan quality indices and clinical factors evaluation

To compare dosimetric parameters, image fusion of iCT and rCT was conducted using the Eclipse treatment planning system based on the same isocenter. Dosimetric parameters (V_105%_, V_103%_, V_100%_, V_98%_, V_95%_, V_90%_, V_50%_: V_x%_ indicates volumes receiving X% of the prescribed dose) were measured in each 3-D FIF plan in the iCT and rCT scans (ECLIPSE™ supports this function of generating each isodose line). We measured V_50%_ because the medial 3-D FIF field edges correspond to the medial junction of V_50%_, as shown in Fig. 1., and we can indirectly measure the irradiated volumes using V_50%_. Thereafter, the dosimetric parameters in the original 3-D FIF plans performed on iCT and those in the identically reproduced 3-D FIF plans performed on rCT were statistically compared.

**Figure 2:**
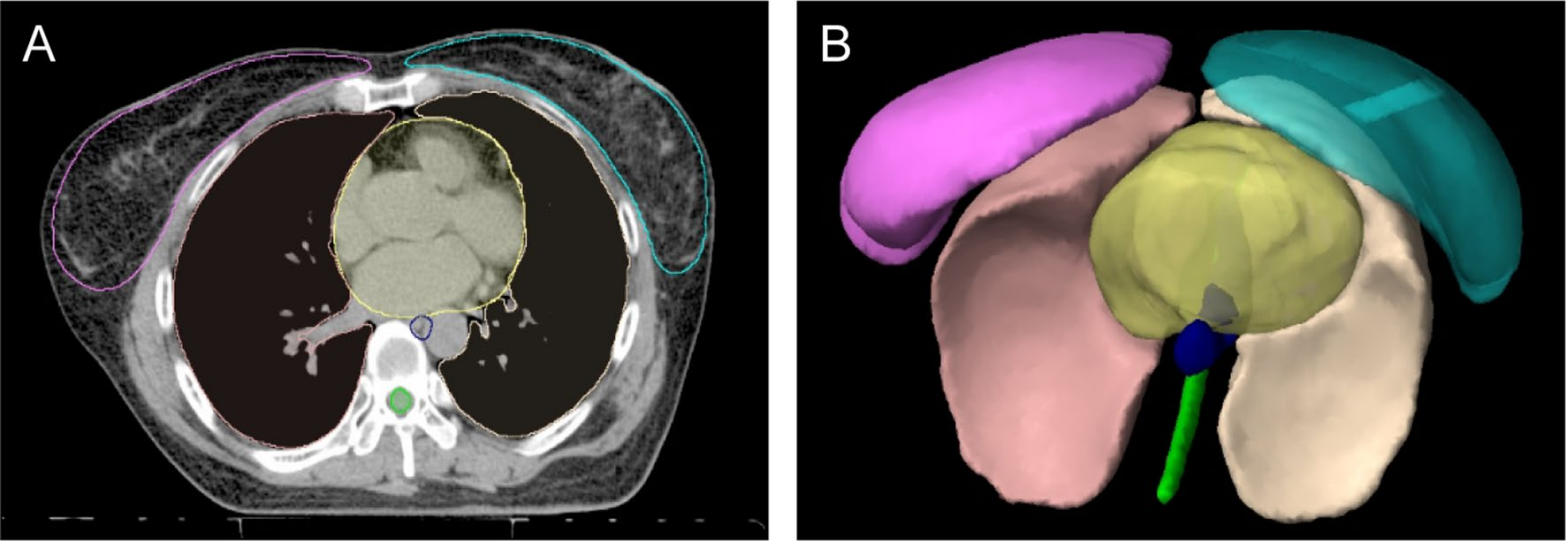
Autocontoured structures in the thorax (produced by Mirada RTx 1.8 and Workflow Box 1.4, Mirada Medical Ltd., Oxford, UK, https://mirada-medical.com/radiation-oncology/) and 3-dimensional rendered images (produced by ECLIPSE™, version 10; Varian Medical Systems, Palo Alto, CA, USA, https://www.varian.com/products/radiosurgery/treatment-planning/eclipse). A: Autocontoured structures in the thorax area, B: 3-dimensional rendered images.

In addition, the following plan quality indices were also acquired^31–33^.

$$ \begin{aligned} & {\text{CI}}\;\left( {{\text{Conformity}}\;{\text{index}}} \right){:}\;{\text{CI}} = {\text{BV}}_{{{95}}} {\text{/PTV}} \\ & {\text{HI}}\;\left( {{\text{Homogeneity}}\;{\text{index}}} \right):\;{\text{HI}} = {\text{D}}_{5} - {\text{D}}_{95} /{\text{D}}_{{\text{p}}} \\ \end{aligned} $$

where BV_95_ represents the volume of the body receiving 95% of the prescribed dose, D_5_ and D_95_ represent the minimum doses to 5 and 95% of the PTV, respectively, and D_p_ represents the prescribed doses. PTV was automatically generated as the breast target volume using Mirada RTx 1.8 and Workflow Box 1.4 (Mirada Medical Ltd., Oxford, UK), a commercial atlas‐based autocontouring product^34^ (Fig. 2).

**Figure 3:**
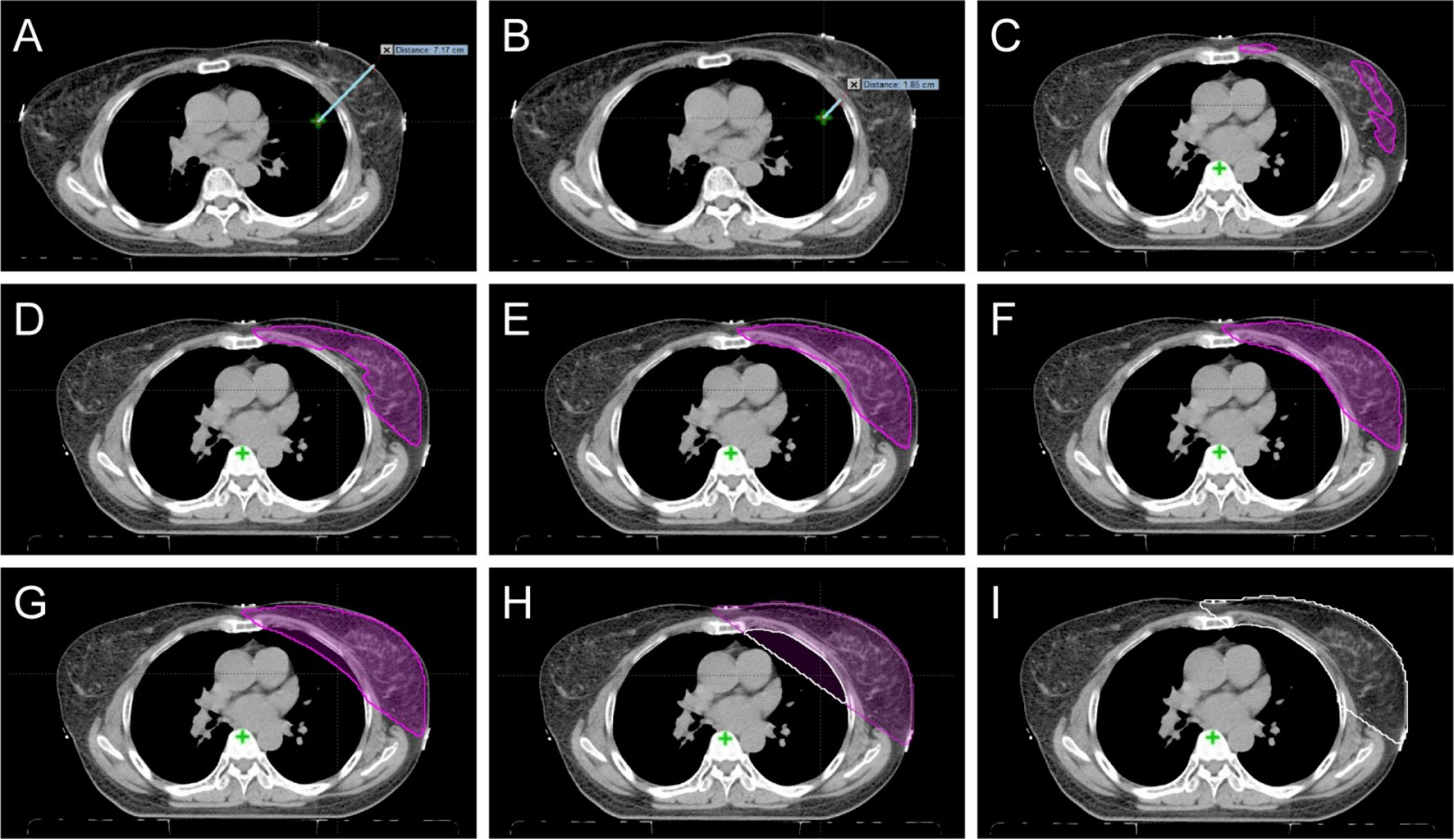
Measurement of clinical and dosimetric parameters. A: Isocenter–Breast axis, B: Isocenter–Lung axis, C: V_103%_, D: V_100%_, E: V_98%_, F: V_95%_, G: V_90%_, H: V_50%_ (pink color) and lung (within RT field) volume (white color), I: Soft tissue (within RT field) volume.

Finally, we measured the following clinical parameters in the iCT and rCT scans: isocenter-breast axis (cm), isocenter-lung axis (cm), soft tissue volumes within RT field (soft tissue volume within 50% isodose lines) (cc), and lung volumes within RT field (lung volumes within 50% isodose lines (cc). The schematic diagrams of how to measure the clinical parameters are shown in Fig. 3.

### Radiation therapy

The entire cohorts were planned to receive 50.4 Gy at 28 fractions of WBI using 6–10 megavoltage photons, followed by a tumor bed boost. Tumor bed boost doses were prescribed according to the surgical margin status with 10–16 Gy at 5–8 fractions of RT. Re-simulation CT was conducted after 45 Gy at 25 fractions of RT.

### Statistical analyses

The statistical analysis was performed using SPSS statistics version 12.0 (SPSS Inc., Chicago, IL). Descriptive statistics and patient demographics were created to show the characteristics of the variables. A normality test was carried out using the Kolmogorov–Smirnov test. The differences in the dosimetric and clinical parameters were compared using a paired *t*-test (parametric) or Wilcoxon rank sum test (non-parametric). The correlation between the variables was assessed using a simple correlation analysis (parametric). A *p*-value of less than 0.05 was considered to be statistically significant.
